# Baiting/Luring Improves Detection Probability and Species Identification—A Case Study of Mustelids with Camera Traps

**DOI:** 10.3390/ani10112178

**Published:** 2020-11-22

**Authors:** Christoph Randler, Tobias Katzmaier, Jochen Kalb, Nadine Kalb, Thomas K. Gottschalk

**Affiliations:** 1 Department of Biology, Eberhard Karls University Tübingen, Auf der Morgenstelle 24, D-72076 Tübingen, Germany; jochen.kalb@gmx.de (J.K.); nadine.kalb@uni-tuebingen.de (N.K.); 2 Department of Regional Management, University of Applied Forest Sciences Rottenburg, Schadenweilerhof 1, D-72108 Rottenburg am Neckar, Germany; tobias.katzmaier@web.de (T.K.); gottschalk@hs-rottenburg.de (T.K.G.)

**Keywords:** attractants, bait, camera traps, glandular scent, marten *Martes*

## Abstract

**Simple Summary:**

Camera traps are now widely used in animal research because they can monitor animals continuously. Nocturnal mammals are particularly difficult to monitor, and identification without cameras would be difficult. However, camera traps can be improved. We here compared two experimental settings to increase detection and images taken of mustelids, mostly martens. Both tuna bait and glandular scents improved the detection and the number of images taken. Both methods were more successful than a control group setting without any attractants.

**Abstract:**

Motion-triggered trail cameras (hereafter camera traps) are powerful tools which are increasingly used in biological research, especially for species inventories or the estimation of species activity. However, camera traps do not always reliably detect animal visits, as a target species might be too fast, too small, or too far away to trigger an image. Therefore, researchers often apply attractants, such as food or glandular scents, to increase the likelihood of capturing animals. Moreover, with attractants, individuals might remain in front of a camera trap for longer periods leading to a higher number of images and enhanced image quality, which in turn might aid in species identification. The current study compared how two commonly used attractants, bait (tuna) and glandular scent (mustelid mix), affected the detection and the number of images taken by camera traps compared to control camera sites with conventional camera traps. We used a before–after control group design, including a baseline. Attractants increased the probability of detecting the target species and number of images. Tuna experiments produced on average 7.25 times as many images per visit than control camera traps, and scent lures produced on average 18.7 times as many images per visit than the control traps.

## 1. Introduction

Motion-triggered trail cameras (hereafter camera traps) are increasingly used in biological research for species inventories or the estimation of species activity. Camera traps can be used to monitor an area without the presence of a human observer. Furthermore, collected data can be assessed by multiple independent researchers and might be used to answer several research questions, including those concerning species inventories [1], patterns of occurrence [2], abundance estimation [3] and species activity patterns [4]. As such, it is not surprising that the number of studies using camera traps significantly increased during the past years [5]. Conducting, for example, species inventories with camera traps can not only help to detect the diversity at a specific site, but also allows a comparison between sites and across seasons, and might even enable researchers to evaluate the impact of human presence on the distribution or behavior of different species [1].

Nonetheless, despite the benefits of camera trapping, medium sized-mammals, especially carnivores, are not easy to monitor, due to their mobility and mostly nocturnal and crepuscular activity. In addition, camera traps frequently fail to detect all individuals of a species, which can bias density estimates. The detection probability is affected by the study design, including the number and position of traps as well as the number of active camera days [6,7,8]. Moreover, the size of the target species and its distance to the camera can affect detection probability [9,10]. Another problem in studies surveying mammals with wildlife camera traps is the insufficient image quality for species or individual recognition, as detected animals are for example very small, too far away or move too quickly in front of the camera trap. This is one limitation of camera traps, because they were originally designed for large game animals, so small, fast moving species may go undetected.

Therefore, attractants, such as food or scents (i.e., substances, materials or devices, which yield odors to attract a target species), can be used to enhance both the likelihood of animals visiting a camera trap and species recognition [11,12,13]. Camera trapping efficiency, for example, increased in a study surveying Iberian lynx (*Lynx pardinus*) after visual and scent lures had been employed [14]. Additionally, free-ranging coyotes (*Canis latrans*) increased their number of visits with increasing odor intensity [15]. However, the number of detected individuals as well as the detection rate depend on the applied attractants [14,16,17]. 

This study aimed to test whether the usage of different attractants, i.e., glandular secretions (scent lure) and tuna (food bait), increase the visiting rate of mustelids at camera traps, with a special focus on stone marten (*Martes foina*) and pine marten (*Martes martes*), which are our target species [18]. These species often occur syntopically [19] and are not easy to identify on camera trap images. Therefore, we used the mustelids as an example to assess the detection and identification of species, with respect to luring/baiting. Furthermore, by applying attractants, the number of captured images might increase, which could increase the likelihood of correct species recognition. This has frequently not been possible in previous studies (e.g., [20]).

## 2. Materials and Methods 

### 2.1. Study Site

The study was conducted on the Spitzberg in Baden-Württemberg (SW Germany). The Spitzberg is located between the city of Tübingen in the east and Rottenburg-Wurmlingen in the west, stretching in length about 6 km and at the widest N–S extension 2 km [21]. The highest point is the Kapellenberg near Wurmlingen, with the highest elevation of 475 m above sea level. The geology of the Spitzberg originates from the Triassic era, about 149 million years ago. On the southern slopes, the forest is almost completely cleared and a large number of terraces with dry stonewalls for winegrowing, cultivated in former times, exist. The largest part is covered by wood, including the heights and the northerly slopes. The forest is characterized mainly by the Scots pine (*Pinea sylvestris*), oak (*Quercus* spec.) and beech (*Fagus sylvatica*). A total of 32 mammal species (excluding bats, Chiroptera) have been reported for the Spitzberg [19,22], including pine and stone marten.

### 2.2. Experimental Methods 

The experiments were performed with a before–after/control–impact design and were based on suggestions by Schlexer (2008 [11]). Two experimental treatments and a control group were applied. First, we used scent lures from the manufacturer Kieferle (Kieferle, Gottmadingen, Baden-Württemberg, Germany) based on mustelids glandular secretions, containing Marten (*M. foina*), weasel (*Mustela* sp.), and polecat (*M. putorius*). The scents were applied according to the instructions given by the manufacturer in *n* = 18 locations, placed >300 m away from each other following previous work (100 m [23], 200 m [24]). During the experiments, we selected periods of weather without heavy rain. However, as the lures are based on oil, rain has only a weak effect on it. In addition, the scents were placed in a tube [23]. To avoid direct human contact with the lure, it was either extracted and applied with a small stick or directly poured onto the ground, trees and stones. Second, at other places (*n* = 18), canned tuna (150 g; “gut & günstig”, manufacturer Edeka, Germany) was chopped into small pieces, extracted with a fork and dispersed around the site. Tuna was placed beneath the bark and in tree and earth holes to avoid the quick exploitation of the food resource. The can was removed to avoid visually attracting and/or injuring species. We used a balanced sampling design with *n* = 18 in each of two experimental treatments and the control group (total *n* = 54 different sites, Table 1). Every site was used only once (i.e., 5 days baseline, 5 days experiment or control). The experimental manipulation (tuna bait, scent lure, control) was randomly distributed. Lures were set 3–4 m directly in front of cameras within their detection zone. At this distance, a mammal the length of a marten (>40 cm, about 1.6 kg) has a high probability of being detected by the camera traps (see, [10]).

The study started with a five-day baseline period without baiting/luring, followed by five days of experimental manipulation. After the baseline period, the digital memory cards were changed, and the cameras and batteries were checked to avoid failure. Immediately afterwards, the experiment was started. The control group camera traps were also visited in a similar manner (e.g., during checking and reading the digital memory cards), but no experimental treatment was applied. This was done to keep possible olfaction impacts caused by humans constant. The five-day period was based on suggestions by Schlexer [11] and findings by Balestrieri et al. [25], who reported that the first marten detection occurred on average (±SD) within 4.2 (±2.8) trap-days. As these studies did not use attractants, we assumed that five days would be long enough for detecting a marten. Some studies did not use a baseline [23,26] because they applied a control group. However, we used an additional baseline for every setting to account for possible pre-treatment differences, such as differences in marten visits prior to the experimental treatments.

### 2.3. Camera Deployment

The study was carried out between 25 April 2018 and 1 September 2018. We used three types of camera traps, which were deployed in a balanced design to apply all experimental variations in all cameras (see Table 1). The distribution of the camera models in the respective treatments was approximately equal (χ^2^ = 1.445, df = 4, *p* = 0.836). The following camera traps were used: SecaCam Raptor, Dörr SnapShot Extra Black 5.0 black (model 204401), Bushnell Natureview (model 119740). For further details on all cameras, see Randler and Kalb [10].

The cameras were placed about 60–80 cm from the camera base to the ground and were slightly skewed towards the ground. After the setup of the cameras, we immediately released the trigger to test functionality. In addition, when coming back to check the digital memory cards and batteries, we approached the camera in a manner that should trigger images. This was done to check if the cameras were still working. We used the highest trigger speed in combination with a series of three images, followed by a 1 min delay. None of the camera traps failed during our study. All camera trap images were checked by the first author. Additionally, images were screened and analyzed by the second author, who was not aware of the applied treatment to reduce potential bias by that knowledge. The data were stored in an Excel sheet with date, time, taxon/species identification, and number of individuals. We coded dichotomously whether a visit had occurred or not.

### 2.4. Statistics

The dependent variables were presence/absence and the number of records/images during a five-day period. For the comparison of dichotomous variables (visit or not) we used a chi-square test and for the comparison of the number of images taken a non-parametric Kruskal–Wallis Test. SPSS version 25 was used for calculations (SPSS Inc., Armonk, NY, USA).

## 3. Results

### 3.1. Detection Probability

**Figure 1:**
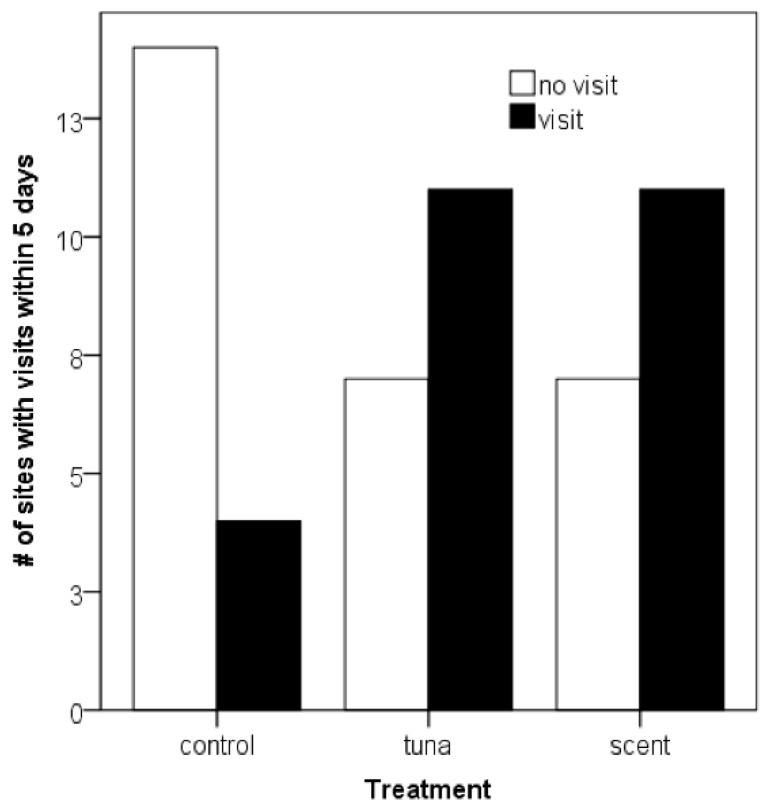
Naïve occupancy (detection probability during the experimental period) of martens at 54 locations dependent on the experimental treatment (tuna or scent) and the control. A total of 18 sites was applied per treatment.

During the baseline period, there was no significant difference in detection probability between the treatments concerning the visits of the target species (χ^2^ = 1.418, df = 2, *p* = 0.492). During the experimental period, the baiting and scents increased the detection probability, i.e., camera traps with either tuna or scent lures recorded more visits during the experimental period compared to control camera traps (χ^2^ = 7.269, df = 2, *p* = 0.026; Figure 1). Mustelid visits were more common at the tuna and scent camera traps. During the experiments, martens were recorded at 11 of 18 sites, both in the tuna- and the scent-lured experiments, while 4 out of 11 detections occurred at the control sites. Thus, detection occurred also at control sites, but the probability increased when scents or tuna were applied.

### 3.2. Number of Images

The number of images taken per five-day period did not differ between the sites during the baseline assessment (Kruskal–Wallis Test, χ^2^ = 1.497, df = 2, *p* = 0.473, Table 2). During the experimental period, there were significant differences between the two treatments and the control (χ^2^ = 9.314, df = 2, *p* = 0.009, Table 2).

Tuna experiments produced on average 7.25 times as many images per visit than the control camera traps (Mann–Whitney U test, Z = −2.653, *p* = 0.017); similarly, scent lures produced on average 18.7 times as many images per visit than the control traps (Mann–Whitney U test, Z = −2.776, *p* = 0.012). There was no difference between the tuna and scent experimental camera traps (Mann–Whitney U test, Z = −0.343, *p* = 0.743). When applying a Bonferroni adjustment (threshold 0.017), both initially significant post-hoc tests remained significant.

### 3.3. Species Identification

We tried to discriminate between different mustelid species and compared the species’ identifiability between the baseline and the experimental period of the attractant experiment. Species identification was higher after the application of attractants compared to the baseline period (Table 3). This was mainly due to the fact of receiving more images, as one can only identify a proportion of the images. However, it is not possible to give the proportion of images that could be identified correctly, because the identification is based on the series of images during an event. For example, on one image the structure of the ears may be seen, while on a second one, the bib marking on the throat is visible, because the marten was turning around.

## 4. Discussion

The results of this study revealed that the use of attractants increased the number of images of the target species per study site, and increased the probability of detection. Camera models usually differ in various aspects including sensor type, trigger speed and detection zone (reviewed in [5]). Therefore, detection success usually varies among camera types and often depends on the size of the target species, its distance from the camera and the speed at which the animals are moving [10,27]. The locations monitored during the experiment did not differ in their mustelid abundance, because there were no significant differences in detection probability between the two treatments and the control during the five-day baseline period. This suggests that using such a baseline method should be applied in further studies based on attractants because it allows both a pre/post evaluation as well as a comparison between sites. This is important because applying only a control/treatment design does not allow one to infer conclusions, as there might be pre-existing differences in mustelid visits prior to the study. 

The detection probability increased in the presence of tuna bait and scent lures compared to the control, as did the number of images. However, the detection probability did not differ between scent and bait, which indicates that martens seem to be equally attracted to both. This is in contrast with previous findings wherein camera traps with fish sauce (anchovy) attracted fewer pine martens, compared to marten lure based mainly on musk [18]. This difference might be caused by differences in bait and lure, because anchovy and tuna are different, and the ingredients of the musk may be different from our lure. Other differences may also lie in the form of application of the tuna baits. Scents are usually based on oils, so they can persist even during rainy weather conditions. Tuna bait, on the other hand, may attract other predators, such as foxes (*Vulpes vulpes*) and badgers (*Meles meles*), that may exploit the food source before martens appear. We used some techniques to avoid this by placing the tuna below stones and behind the bark of trees. Lastly, the different results might be caused by the fact that we could not always differentiate between pine martens and stone martens. Even though the trophic niches of these two species greatly overlap, they show quantitative differences in their food consumption [28]. It would be beneficial to identify lures and baits that only attract the marten species of interest. However, this might be difficult with carnivores in general, but is a worthy research objective. One alternative might be looking at the automated delivery of a bait so that it is not always devoured by non-target carnivore species. However, more studies are needed to investigate if such attractants exist and how to apply them in order to collect reliable research data. A final comparison between tuna bait and scent lures comes down to ease of use and cost. Canned tuna is cheaper and easier to apply next to camera traps than the scents. Since both attractants worked equally well in our setup, one might choose to only use tuna baits to reduce the cost and time effort for monitoring marten activity. However, this only holds if tuna remains inaccessible by other predators and thus remains viable as a lure.

## 5. Conclusions

Based on our results, combining camera traps and either bait or scent is an effective way to monitor the presence and behavior of mustelids. However, as the visits between unbaited and baited camera traps differed, the relative abundance of a species might be significantly influenced by attraction methods, and thus does not reflect the natural behavior as would be detected by unbaited traps. The huge SD for the scent lure suggests that animals are hanging around longer and recording more photos, and this could help with identification. 

## Figures and Tables

**Table 1:** Overview of the cameras used in the study.

Camera Model	Treatment			
	Control	Tuna	Scent	Total
Dörr	6	4	3	13
Natureview	1	1	1	3
Secacam	11	13	14	38
Total	18	18	18	54

**Table 2:** Mean number of images per site (within the 5-day period) in two different treatments (tuna, scent) and the control sites during baseline and the experimental period.

	Images Baseline			Images Experiment		
Treatment	Mean	SD	Median	Mean	SD	Median
Control	0.61	1.335	0.00	0.72	1.602	0.00
Tuna	0.44	1.149	0.00	5.22	6.54	3.0
Scent	1.17	2.176	0.00	13.44	24.108	3.0

**Table 3:** Number of identified mustelid species dependent on the experimental period.

Species	Baseline	Experiments
Stone marten	2	3
Pine marten	1	6
European polecat	0	1
*Martes* spp.	4	2
Total visits	7	12
